# Endocyclophotocoagulation combined with phacoemulsification in surgically naive primary open-angle glaucoma: three-year results

**DOI:** 10.1038/s41433-021-01734-4

**Published:** 2021-09-15

**Authors:** Timothy E. Yap, Piero Zollet, Salman Husein, Mohammad M. M. Murad, Sally Ameen, Laura Crawley, Philip A. Bloom, Faisal Ahmed

**Affiliations:** 1 The Western Eye Hospital, Imperial College Healthcare NHS Trust (ICHNT), London, UK; 2 The Imperial College Ophthalmology Research Group (ICORG), Imperial College London, London, UK; 3 Department of Ophthalmology, Università Vita-Salute San Raffaele, Milan, Italy; 4 Research Institute of Ophthalmology, Giza, Egypt

**Keywords:** Optic nerve diseases, Surgery

## Abstract

**Objective:**

To assess the safety and efficacy of endocyclophotocoagulation with phacoemulsification (phaco-ECP) in surgically naive, primary open-angle glaucoma (POAG).

**Methods:**

A retrospective case series of patients undergoing phaco-ECP between 2007 and 2017 at a single centre in London, UK. The primary outcome was intraocular pressure (IOP). Secondary outcomes were visual acuity, visual field global indices, topical medications and surgical complications. Failure criteria were: (1) IOP > 21 mmHg or <20% reduction at two consecutive visits, (2) IOP <5 mmHg and (3) further IOP-lowering surgery.

**Results:**

Eighty-three eyes from 83 patients were eligible. Pre-operatively, mean IOP (±SD) was 18.4 ± 5.2 mmHg. The mean number of topical agents (±SD) was 2.7 ± 0.9. Mean IOP (±SD) significantly reduced to 14.3 ± 4.7 at 1 year, 14.1 ± 4.0 at 2 years and 13.6 ± 3.7 at 3 years (*p* < 0.0001). Topical medications were significantly reduced to 1.3 ± 1.2 at 1 year, 1.7 ± 1.2 at 2 years and 1.8 ± 1.3 at 3 years (*p* < 0.0001). Annual IOP ‘survival’ was 70%, 54% and 45% at year 1, 2 and 3, respectively. Complications included uveitis (6%), macular oedema (2%), IOP spikes (1%) and corneal decompensation (1%) with no episodes of hypotony or retinal detachment. One patient underwent filtration surgery within 3 years (1%).

**Conclusion:**

Phaco-ECP facilitates significant IOP lowering and reduction of medication burden in surgically naive POAG requiring cataract extraction. The procedure is relatively safe and without the use of implants and their associated risks.

## Introduction

Glaucoma is a progressive optic neuropathy with the potential to cause blindness. It is characterised by ‘cupping’ of the optic nerve head due to the loss of retinal ganglion cells and their associated axonal fibres that converge at the optic disc [1]. The most important and only modifiable risk factor for the development and progression of glaucoma is raised intraocular pressure (IOP) [2–5]. IOP can be lowered with topical or systemic medications, laser or surgical procedures.

Laser ablation of the ciliary epithelium in order to reduce aqueous humour production can be achieved transsclerally or endoscopically [1]. Transscleral cyclophotocoagulation is traditionally reserved as a last resort IOP-lowering procedure in patients with advanced glaucoma due to the potential risks of hypotony, inflammation and visual loss [6]. More recently, MicroPulse diode transscleral cyclophotocoagulation has been introduced [7] with consistent results and a good safety profile. In contrast to the transscleral approach, endoscopic cyclophotocoagulation (ECP) allows the titration of diode laser treatment to the ciliary epithelium by direct endoscopic view. First described in 1992 [8], this technique delivers more predictable results with a favourable safety profile, thus being suitable for a wider range of patients. Further benefits include the avoidance of damage to surrounding tissues and sclera, patient acceptability and the availability of reusable probes.

For surgically naive patients with cataract and suboptimally controlled primary open-angle glaucoma (POAG), there are several factors involved when choosing the appropriate treatment. The clinician is likely to consider the immediate improvement in vision-related quality of life from cataract extraction, versus the urgency or degree of IOP lowering required. Furthermore, patient- and eye-related factors can also make certain surgery in specific cases higher risk [9]. Minimally or micro-invasive glaucoma surgery (MIGS) has proven popular for when filtration surgery is thought unnecessary, for those with drug intolerance, and those struggling with medication compliance [10]. For these patients with cataract, a resurgence of interest in ECP has arisen.

Prospective 2-year phacoemulsification-ECP (phaco-ECP) outcomes have shown significantly greater reduction in IOP and topical medication use compared to phacoemulsification alone (non-randomised) [11], with retrospective studies showing similar results [12, 13]. Mean IOP reduction has been reported between 7.1 mmHg [14] and 10.9 mmHg [15] at 2 years in retrospective studies, and 2.1 mmHg prospectively. Only two studies have reported outcomes of 3 years in duration, however not specifically in relation to open-angle glaucoma [12] or surgically naive eyes [16]. In the study most similar to ours, Smith et al. reported a mean reduction of 4.7 mmHg and 58.3% ‘failure’ at 3 years, with failure defined by an IOP higher than 21 mmHg or lower than 6 mmHg, IOP not reduced by 20% from baseline at annual intervals or further procedures to reduce IOP [16]. Our study reports 3-year outcomes of patients following phaco-ECP, exclusively in surgically naive POAG eyes.

## Methods

### Study design

This study was a retrospective case series of combined phaco-ECP in surgically naive eyes of patients with POAG. Patients who had phaco-ECP between May 2007 and March 2017 were identified via a database search. This study complied with the tenets of the Declaration of Helsinki, and it was given local regulatory approval. The minimum sample size of 47 eyes was based on a minimum detectable effect of 3 mmHg, a standard deviation of 4.8 mmHg, with alpha = 0.05 and 85% power [16].

### Inclusion criteria

Eligible patients were at least 18 years of age and diagnosed with POAG (of any severity). Included eyes were surgically naive prior to phaco-ECP, with comprehensive ECP treatment indicated for suboptimally controlled IOP. In all cases, between 270 and 360 degrees of ECP was actually delivered, as recorded in the post-operative notes. Only the first eye to receive phaco-ECP per patient was included in the study.

### Exclusion criteria

Patients were excluded if they had previously undergone laser treatments including diode laser cyclophotocoagulation, laser trabeculoplasty or laser peripheral iridotomy in the study eye. Patients with normal-tension glaucoma, or any secondary glaucoma including pseudoexfoliation or pigment dispersion syndrome were excluded.

### Surgical technique

Informed consent for surgery was given by all patients prior to their operation. Surgeries were performed by glaucoma specialist consultants and glaucoma fellows with experience in phaco-ECP. Topical, local or intracameral anaesthesia was used as needed, with a few patients also requiring general anaesthesia. Topical anaesthesia was delivered with 0.5% proxymetacaine, local anaesthesia with sub-Tenon’s 2% lidocaine and intracameral anaesthesia using 1% unpreserved lidocaine. Sub-Tenon’s blocks were carried out under topical anaesthesia via a 2 mm conjunctival incision, 8 mm from the inferonasal limbus. A 19-gauge sub-Tenon’s cannula was then used to inject 5 ml of 2% lidocaine into the sub-Tenon’s space. Phacoemulsification and insertion of an intraocular lens implant was carried out first. An additional incision was made 180 degrees away from the main incision using a 2.4 mm keratome blade. An ophthalmic viscosurgical device was used to widen the ciliary sulcus in order to gain a view of the ciliary body processes. An endoscopic image was acquired by inserting the probe into the main incision, with the view adjusted to show approximately six ciliary processes at a time. Circumferential (between 270 and 360 degrees) 810 nm diode laser was applied using the shrinkage and whitening of the processes as a visible endpoint. Power was initially set at 0.25 W, and titrated up to 0.5 W according to effect. Intracameral cefuroxime (1.0 mg/0.1 ml) and dexamethasone (0.1 ml of 3.3 mg/ml preservative-free) were injected following ECP. Wounds were routinely closed using hydration, with sutures required in the minority of cases. Patients received standardised post-operative drops composed of 1 week of 0.5% chloramphenicol antibiotic four times daily, and a reducing regimen of 0.1% dexamethasone starting at six times daily and typically tapered by one drop each week. Pre-operative topical hypotensive agents were continued until post-operative clinic review, followed by titrated reduction. The rate and magnitude of this reduction was conducted on a case-by-case basis according to target IOP.

### Outcome measures

The primary outcome measure was IOP. Secondary outcomes were best-corrected visual acuity (BCVA), visual field mean deviation (MD) and pattern standard deviation (PSD), number of topical ocular hypotensive agents and complications. The presence or absence of predefined complications was noted during data collection. These were defined as hypotony (IOP < 5 mmHg), IOP spike (IOP > 30 mmHg within a week of surgery), anterior uveitis (fibrinous anterior uveitis or ‘rebound’ anterior uveitis requiring an increase in steroid therapy), macular oedema, choroidal effusion, retinal detachment and corneal decompensation, in addition to any other complications that may have occurred.

### Survival analysis

Survival analyses were performed six times based on three different IOP criteria; ‘A’, ‘B’ and ‘C’, both with and without respect to topical medication use. Criteria ‘A’ were defined as the presence of one or more of the following failure criteria: (1) IOP > 21 mmHg or <20% reduction from baseline at two consecutive visits, (2) IOP < 5 mmHg at any visit or (3) further IOP-lowering surgery. Criteria ‘B’ included an upper IOP threshold of >18 mmHg, and criteria ‘C’ included an upper IOP limit of >15 mmHg. The topical agent criterion was then added to each of the IOP criteria, whereby ‘failure’ was reached if a greater number of topical agents (including individual drugs in combination preparations) were being used post-operatively (at least 90 days after surgery).

### Data collection

A review of clinical notes was performed for the eligible patients. Baseline demographics were collected including age, ethnicity and gender. Ophthalmic data at each visit including IOP, BCVA, visual field MD, number of topical and systemic glaucoma medications, post-operative complications and number and type of subsequent laser procedures and surgeries were recorded. Reasons for loss to follow-up were collected. Any visual acuity readings recorded in Snellen were converted to Logarithm of the Minimum Angle of Resolution (LogMAR) according to published guidelines [17]. Medications were assessed per pharmacological agent, including the individual agents within combination preparations.

### Statistical methods

The data of eligible patients were exported onto spreadsheets (Microsoft Corp.) and the statistical analyses performed using R (R Foundation for Statistical Computing, Vienna, Austria). Graphs were produced using GraphPad Prism (version 7.0c, GraphPad Software, Inc.). D’Agostino-Pearson omnibus normality test was used to test for the presence or absence of a normal distribution in the data. Comparisons between preoperative and post-operative data were performed by using a one-way ANOVA with correction for multiple comparisons (Dunnett’s test), with a *p* value <0.05 considered statistically significant. The final reading per year was included in the annual summary data, with the last pre-operative observation carried forwards in the case of further surgery. For survival analysis, right censoring took place in cases of death or loss to follow-up. Kaplan–Meier plots were used to illustrate ‘survival’.

## Results

### Demographics and baseline parameters

Table 1 presents a summary of demographics and baseline characteristics.

**Table 1:** Patient demographics and baseline characteristics.

*Patient demographics*	
Patients	83
Eyes	83
Age at surgery (mean ± SD, years)	76.1 ± 12.0
Males:Female	57%:43%
Ethnicity, *n*	
Caucasian	30 (36%)
African/Afro-Caribbean	28 (34%)
Asian	8 (10%)
Unknown	17 (20%)
*Baseline characteristics*	
BCVA (mean ± SD, LogMAR)	0.59 ± 0.50
IOP (mean ± SD, mmHg)	18.4 ± 5.2
Mean deviation (mean ± SD, dB)	–13.1 ± 9.4
Mild (MD > –6 dB)	26%
Moderate (–6 dB > MD > –12 dB)	19%
Severe (MD < –12 dB)	54%
Pattern standard deviation (mean ± SD, dB)	6.2 ± 3.3
Number of glaucoma agents (mean ± SD)	2.7 ± 0.9

*BCVA* best-corrected visual acuity, *IOP* intraocular pressure, *MD* mean deviation.

### Intraocular pressure

**Fig. 1:**
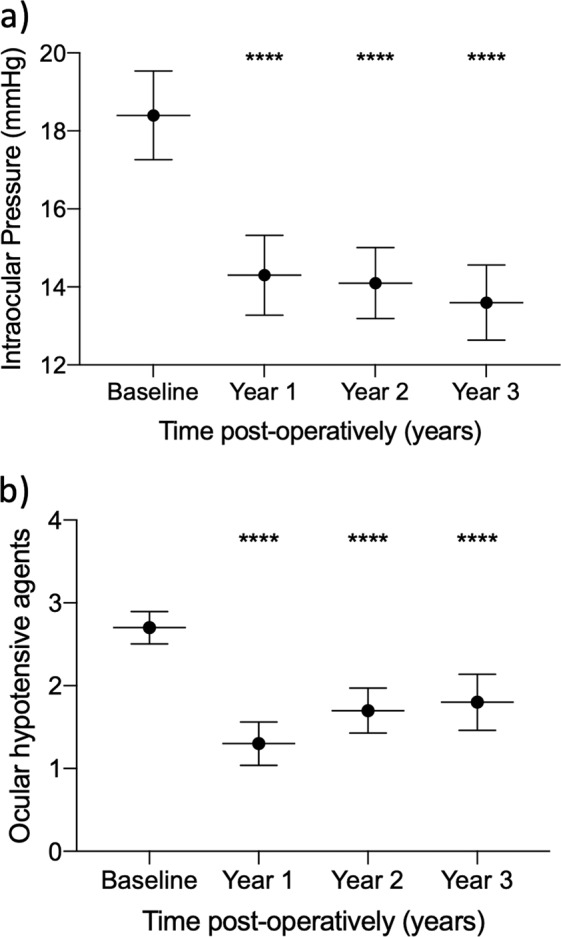
Intra-ocular pressure change over time. Intraocular pressure outcomes showing **a** mean IOP (±95% CI) per year and **b** mean number of ocular hypotensive agents (±95% CI) per year. Significant change from baseline in both outcomes was seen at all timepoints, corrected for multiple comparisons (****p < 0.0001). IOP intraocular pressure.

Pre-operatively, mean IOP (±SD) was 18.4 ± 5.2 mmHg. Post-operatively, mean IOP (±SD) was 14.3 ± 4.7 1 year after surgery, 14.1 ± 4.0 at 2 years and 13.6 ± 3.7 mmHg at 3 years. These values were significantly lower than pre-operatively at all timepoints (*p* < 0.001). This equated to a mean reduction of 4.1 ± 5.6 mmHg at 1 year (22%), 4.3 ± 5.9 mmHg at 2 years (23%) and 4.8 ± 5.6 mmHg at 3 years (26%) (Fig. 1, Table 2 and Supplementary Fig. 1). The characteristics of patients lost to follow-up and those with missing annual data, in addition to reasons for loss to follow-up are presented in the Supplementary table.

**Table 2:** Annual summary measures of all study eyes (final visit per year).

Parameter	Baseline (*n* = 83)	1 year (*n* = 83)	2 years (*n* = 77)	3 years (*n* = 59)
IOP change, mmHg (mean ± SD)	18.4 ± 5.2	–4.1 ± 5.6	–4.3 ± 5.9	–4.8 ± 5.6
Percentage IOP reduction	–	22%	23%	26%
Change in ocular hypotensive agents (mean ± SD)	2.7 ± 0.9	–1.4 ± 1.4	–1.0 ± 1.4	–0.9 ± 1.5
Best-corrected visual acuity, LogMAR (mean ± SD)	0.59 ± 0.50	0.30 ± 0.41	0.26 ± 0.44	0.26 ± 0.39
Patients on no medications, no further surgery	0%	35%	22%	25%
Visual field, dB (mean ± SD)				
MD	–13.1 ± 9.4	–11.7 ± 8.4	–13.5 ± 9.5	–13.1 ± 9.7
PSD	6.2 ± 3.3	6.6 ± 3.9	6.5 ± 3.3	6.3 ± 3.5

Last pre-operative observation was carried in the case of subsequent filtration surgery (*n* = 1).

*IOP* intraocular pressure, *MD* mean deviation, *PSD* pattern standard deviation.

### Survival analysis

**Fig. 2:**
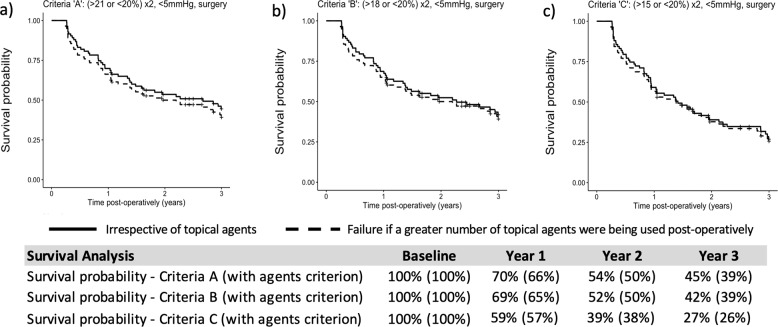
Survival analysis. Kaplan–Meier survival curves showing survival according to the following failure criteria (at least one): **a** Criteria ‘A’: IOP > 21 mmHg or <20% reduction at two consecutive visits, IOP < 5 mmHg, or further IOP-lowering surgery. **b** Criteria ‘B’: upper IOP limit >18 mmHg, **c** Criteria ‘C’: upper IOP limit >15 mmHg. Solid line: failure irrespective of topical agents. Dashed line: failure if a greater number of topical agents were being used post-operatively (at least 90 days after surgery).

Survival according to failure criteria ‘A’ (not including topical agents criterion) was 70% at 1 year, 54% at 2 years and 45% at 3 years. Including the agents criterion, survival according to criteria ‘A’ was 66% at 1 year, 50% at 2 years and 39% at 3 years. Full results for failure criteria A–C excluding and including the topical agents criteria are displayed in Fig. 2a–c.

### Number of ocular hypotensive agents

Pre-operatively, the mean number of topical ocular hypotensive agents ± SD (separate agents counted within combination preparations) was 2.7 ± 0.9 agents. Post-operatively, mean ± SD was 1.3 ± 1.2 agents at 1 year, 1.7 ± 1.2 agents at 2 years and 1.8 ± 1.3 agents at 3 years. This equated to a mean reduction of 1.4 ± 1.4 agents at 1 year, 1.0 ± 1.4 agents at 2 years and 0.9 ± 1.5 agents at 3 years. This was a significant reduction from baseline at all timepoints (*p* < 0.0001). There were no patients off glaucoma medications at baseline, with the percentage of patients off glaucoma medications and having had no further surgery being 35% at 1 year, 22% at 2 years and 25% at 3 years (Table 2). No systemic glaucoma medications were used in this series of cases.

### Visual acuity

Pre-operatively, mean BCVA ± SD was 0.59 ± 0.50 LogMAR (6/24 Snellen). Post-operatively, mean BCVA ± SD was 0.30 ± 0.41 LogMAR at 1 year (6/12 Snellen), 0.26 ± 0.44 LogMAR (6/12 Snellen) at 2 years and 0.26 ± 0.39 LogMAR (6/12 Snellen) at 3 years. BCVA was significantly improved compared to baseline at each timepoint (*p* < 0.0001) (Table 2).

### Visual field

Mean visual field MD at baseline was –13.1 ± 9.4 dB. Post-operatively, MD was –11.7 ± 8.4 dB at 1 year, –13.5 ± 9.5 dB at 2 years and –13.1 ± 9.7 dB at 3 years. Mean visual field PSD was 6.2 ± 3.3 dB at baseline, and 6.6 ± 3.9 dB at 1 year, 6.5 ± 3.3 dB at 2 years and 6.3 ± 3.5 dB at 3 years post-operatively. There was no significant change from baseline in either parameter (*p* > 0.05) (Supplementary Fig. 2).

### Complications and additional procedures

Complications included five episodes of granulomatous or ‘rebound’ anterior uveitis (6%), two cases of macular oedema (2%), one IOP spike (1%) and one corneal decompensation in a patient with pre-existing herpetic disease (1%). Three of the cases of anterior uveitis (as per the definition in the methods section) resolved with topical therapy, with the other two cases requiring subconjunctival steroid. Resolution in these cases was achieved between 2 and 4 months following surgery, with corrected visual outcomes of 0–0.3 LogMAR (6/6–6/12 Snellen). Both cases of post-operative macular oedema were complicated by the worsening of pre-existing conditions; one eye had worsening of epiretinal membrane leading to a best-corrected vision of 0.6 LogMAR (6/24 Snellen), with the other complicated by pre-existing diabetic maculopathy requiring intravitreal therapy resulting in a BCVA of 0.5 LogMAR (6/18 Snellen). All IOP spikes resolved with topical therapy. The case of corneal decompensation resolved back to baseline with topical antiviral therapy, with a VA of 0.2 LogMAR (6/9.5 Snellen). No retinal detachments, episodes of hypotony, choroidal effusions or other, non-predefined complications were observed (Table 3).

**Table 3:** Complications and additional treatments delivered.

ECP complications, *n (%)*	
Anterior uveitis	5 (6)
Macular edema	2 (2)
IOP spike	1 (1)
Corneal decompensation	1 (1)
Hypotony	0
Retinal detachment	0
*Additional therapies and re-treatment, n (%)*	
Trabeculectomy	1 (1)
Years before additional surgery	2.7

*ECP* endocyclophotocoagulation, *IOP* intraocular pressure.

In our study, one further IOP-lowering surgery (1%) was performed within 3 years of phaco-ECP (trabeculectomy with mitomycin C). This was performed at 2.7 years post-operatively (Table 3).

## Discussion

This study is unique in reporting phaco-ECP outcomes exclusively in surgically naive, suboptimally controlled POAG, whilst including all severities of visual field loss. The results demonstrate that phaco-ECP can facilitate IOP reduction and reduced topical therapy use compared to baseline over a 3-year period. Furthermore, the novel failure criteria comparing topical agent use with baseline comprehensively capture both high IOP, and that which has required treatment.

At the 3-year timepoint, mean IOP was reduced by 26%. Although ‘survival’ rates were reduced to 45% by the end of the third post-operative year (Criteria A), the low rates of filtration surgery during this period were encouraging, with the agents criterion demonstrating the lack of overwhelming medication use compared to baseline. The paradoxical reduction in annual IOP with increasing failure rates in our data is explained by the contrasting way these data were collected. For the annual summary measures, only the final measurement per year was captured. Evidence for treatment initiation in reaction to intercurrent rises in IOP can be observed in the steady rise of glaucoma treatments after year 1. No significant deterioration in visual field was observed even in the absence of inclusion criteria based on disease severity. Although other studies have been limited to ‘mild-moderate glaucoma’, the reporting of visual field indices is noticeably lacking [11, 12, 14]. Bartolomé et al. [13], however, did report a baseline MD similar to that of the patients in this study (–13.4 vs –13.1 dB).

Although phaco-ECP has been shown to facilitate IOP reduction, the impact of cataract extraction alone must be considered. Francis et al. [11] performed a prospective, non-randomised study comparing phaco-ECP to phacoemulsification alone, with follow-up of 3 years. They reported a mean IOP reduction of 13.6 ± 15.1% at 3 years after phaco-ECP in comparison to 5.1 ± 10.4% in the phacoemulsification only group (*p* = 0.003). A 12-month retrospective study from Bartolomé et al. (not restricted to surgically naive eyes) also reported significantly greater IOP reduction following phaco-ECP, however with a significantly lower mean pre-operative IOP in the phacoemulsification only group [13]. The results of a similar retrospective study by Siegel et al. [12] highlighted the trade-off between IOP lowering and medication use; although their results showed medication reduction, consistent IOP lowering was not found. It is interesting to note that despite the lack of IOP-lowering, Siegel et al. were able to declare phaco-ECP superior due to the study population being pre-operatively defined as ‘well-controlled’. This acts to highlight both the opportunistic manner in which ECP can be combined with cataract surgery, and the wish of many patients to be free of eyedrop administration.

Other retrospective case series have reported IOP reductions lower [18–20], similar [16, 21] and higher [14, 15, 22] than the results in this study, with most observing a reduction in efficacy through time. This may be due to a partial recovery of the ciliary epithelium [23] or a homeostatic response in the balance of aqueous production and drainage. However, direct comparisons between publications are challenging for three reasons. First, inclusion criteria vary between studies in terms of disease phenotype, surgical naivety, whether IOP was within target range prior to surgery, and disease severity. Second, failure criteria differ between publications. Third, the trade-off between IOP and medication mentioned above may vary on an institutional, study cohort or individual patient level. For example, Smith et al. [16] found a mean IOP reduction of 25% at 3 years (26% in our study), but no significant reduction in medication use. Lindfield et al. also published a 2-year retrospective case series reporting a 33% IOP reduction at 2 years (compared with 23% in our study), however no change in topical medication use [14]. The successful finding of simultaneous IOP reduction in combination with reduced medication use in our study may be a consequence of inclusion criteria, varying levels of IOP/eyedrop trade-off or surgical nuance. To further extricate the factors in this trade-off, we believe the dual-level failure criteria unique to this study comprehensively detect both raised IOP, or an excess of medications required to treat it, allowing success to be assessed for a range of target IOPs and eyedrop tolerance.

ECP is viewed by some as the original ‘MIGS’ procedure to lower IOP following cataract surgery. In comparison, trabecular bypass implants (iStent, Glaukos, Laguna Hills, California, USA) have been shown to provide 9% IOP reduction with a single implant and 27% with a double implant, compared to 4.7% with phacoemulsification alone at 12 months [24]. Combinations with ECP have also been assessed, with the combined double trabecular bypass stent procedure (‘ICE2’) providing 35% IOP reduction at 12 months compared to phaco-iStent alone (21% reduction) [25]. A cost analysis of iStent conducted by Tan et al. [26] reported that although savings made from reduced eyedrop burden may be quick to be found in comparison to branded eyedrops, such savings would be longer to materialise in comparison with generic agents due to the significant per-procedure expense of such devices. In contrast, a one-off financial investment for an ECP machine that uses reusable probes may be a more cost-effective long-term strategy for patients already undergoing cataract surgery, with continually reducing per-procedure expense [27, 28].

Recent comparisons with filtration surgery combined with phacoemulsification are not possible due to changing trends in surgical management. Gayton et al. [29] published a prospective, randomised study in 1999 comparing phaco-ECP and phaco-trabeculectomy with 24 months of maximum follow-up. The phaco-ECP group achieved IOP control (<19 mmHg) in 30% of cases at year 2, compared with 40% in the phaco-trabeculectomy group. Notably, phaco-ECP required a lower number of reinterventions (10% vs 14%). Several conjunctival-sparing alternatives to trabeculectomy such as ab interno trabeculectomy [30] and canaloplasty [31] have been developed leading to comparisons [32] and combinations [33, 34] with phaco-ECP, with phaco-ECP plus dual blade ab interno trabeculectomy (PEcK) providing an IOP reduction of 5.1 ± 4.4 mmHg at 12 months [34]. However, such techniques are yet to be widely taken up, perhaps owing to the required technical skills or a differential safety profile. However, phaco-ECP complications in our study are low, with data published from 5824 eyes by the ECP Collaborative Study Group reporting IOP spikes (14.5%), haemorrhage (3.8%), serous choroidal effusion (0.36%), retinal detachment (0.27%) and hypotony (0.12%) with no chronic inflammation or endophthalmitis reported [35].

In conclusion, our study shows that phaco-ECP is beneficial for a range of surgically naive suboptimally controlled POAG patients requiring cataract extraction. This is especially relevant to those who could also benefit from fewer topical agents, however with increasing failure rates at 3 years post-operatively. Given the ab interno approach and minimal disruption to tissue, ECP should be considered in parallel with MIGS devices when deciding to combine cataract extraction with an IOP-lowering procedure. Future studies should carefully consider whether to include only surgically naive eyes or those undergoing second-line procedures, along with whether their study population has suboptimal IOP control (and thus are likely to begin with higher starting pressures) or whether reduction of topical medication alone is considered successful. Differences in study populations, failure criteria and thresholds for topical therapy use, along with a lack of prospective randomised trials and short follow-up periods are hurdles in the interpretation and comparison of studies of this nature.

### Summary

#### What was known before

- Phacoemulsification-ECP provides IOP lowering or reduction of topical therapy use in heterogenous glaucoma populations.

#### What this study adds

- In a unique, exclusively surgically naive POAG population, ECP combined with phacoemulsification provides IOP lowering and a reduction in the use of topical therapy associated with an excellent safety profile, and therefore should be considered as an alternative to MIGS devices.

## Supplementary information

Supplementary Figure 1

Supplementary Table

Supplementary Figure 2
