# Supplementary figures and images for: Endocyclophotocoagulation combined with phacoemulsification in surgically naive primary open-angle glaucoma: three-year results
Source: Eye (Lond). 2021 Sep 15;36(10):1890–5. doi: 10.1038/s41433-021-01734-4 (PMC9499941; doi:10.1038/s41433-021-01734-4)

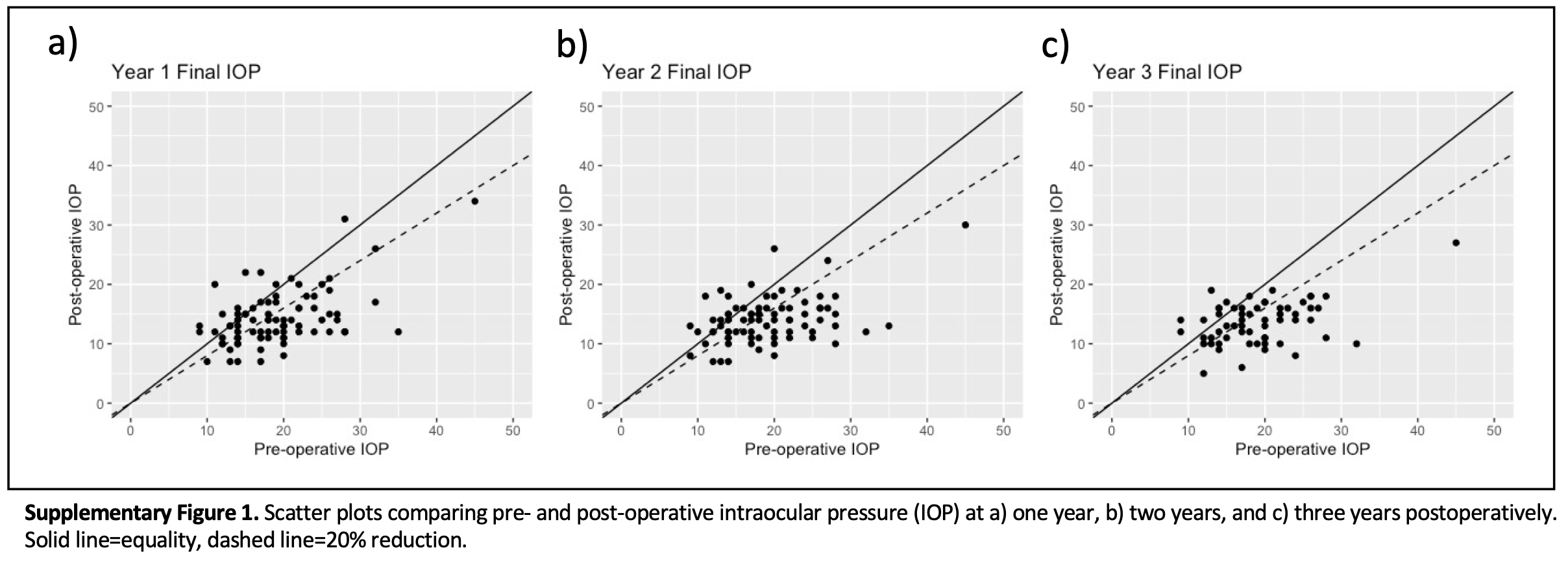

Supplement: Supplementary file 1 — Supplementary Figure 1 [file 41433_2021_1734_MOESM1_ESM.tif]

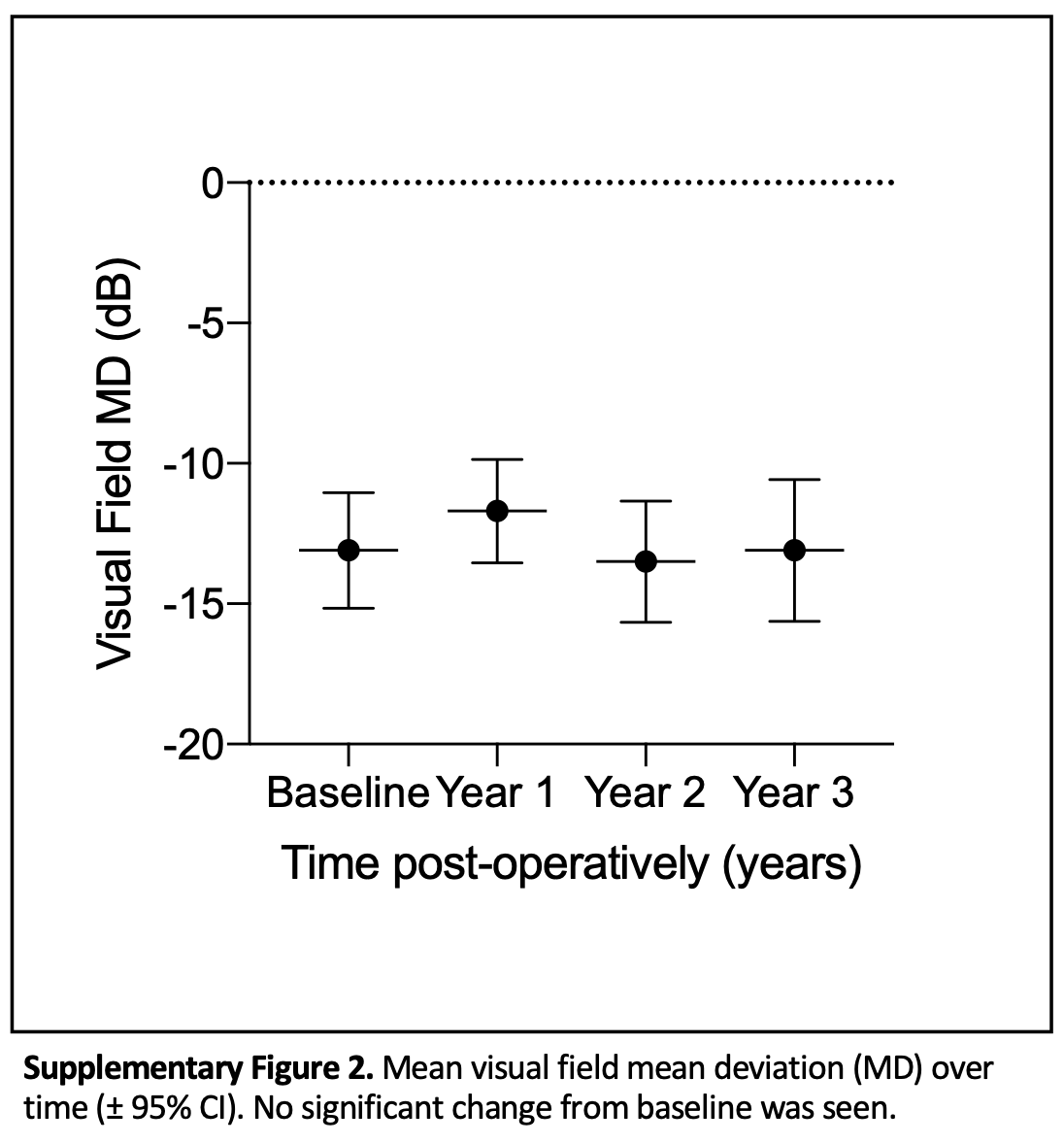

Supplement: Supplementary file 3 — Supplementary Figure 2 [file 41433_2021_1734_MOESM3_ESM.tif]
